# Refractory hypertension secondary to renal artery stenosis with a honeycomb-like structure

**DOI:** 10.1186/s12872-021-02428-1

**Published:** 2021-12-20

**Authors:** Cheng Chen, Ying Zhang, Da Yin, Yan Liu, Yunpeng Cheng, Yan Lu, Yinong Jiang, Wei Song

**Affiliations:** 1 Department of Cardiology, The First Affiliated Hospital of Dalian Medical University, No. 222 Zhongshan Road, Zhongshan District, Dalian, Liaoning Province China; 2 State Key Laboratory of Cardiovascular Disease, Fuwai Hospital, National Center for Cardiovascular Diseases, Chinese Academy of Medical Sciences and Peking Union Medical College, Beijing, China

**Keywords:** Secondary hypertension, Renal artery stenosis, Honeycomb-like structure, IVUS, Case report

## Abstract

**Background:**

A honeycomb-like structure (HLS) is a rare abnormality characterized by a braid-like appearance. Angiograph and intravascular examination, including coherence tomography and intravascular ultrasound (IVUS), can further confirm the multiple intraluminal channels or honeycomb structure, which can also be described as looking like ‘swiss cheese’, a ‘spider web’ or a ‘lotus root’. Previous studies have mostly reported this abnormality in coronary arteries, with a few cases in renal arteries. More information about the characteristics and development of HLS is needed.

**Case presentation:**

A 69-year-old Han man with resistant hypertension received abdominal enhanced computerised tomography and was revealed to have left renal artery stenosis with the possibility of left renal infarction. Renal artery angiography confirmed a 95% stenosis located in the proximal segment of the left renal artery, and the middle segment was blurred with multi-channel-like blood flow. Further IVUS was performed and identified multiple channels surrounded by fibrous tissue. It was a rare case of HLS in the renal artery secondary to the thrombus, with organisation and recanalisation. Balloon dilatation and stent implantation at the proximal segment of the left renal artery were performed successfully. Blood pressure was well controlled after the procedure.

**Conclusions:**

The IVUS findings are helpful for forming interventional therapeutic strategies for HLS lesions in the renal artery.

**Supplementary Information:**

The online version contains supplementary material available at 10.1186/s12872-021-02428-1.

## Background

A honeycomb-like structure (HLS) was first described by Terashima et al. in a 26-year-old man with a history of Kawasaki disease at the proximal segment of the left anterior descending artery (LAD) [1]. This abnormality is rare in that its aetiology and characteristics are still unclear. In addition, compared to work on coronary arteries, studies concentrating on HLS involved in renal arteries are relatively scarce [2, 3]. Therefore, we herein describe the case of a 69-year-old man with HLS along with renal artery stenosis, secondary hypertension and suspected renal infarction treated with interventional treatment. To our knowledge, this is the first case of HLS on the basis of atherosclerosis in the renal artery.

## Case presentation

**Fig.1:**
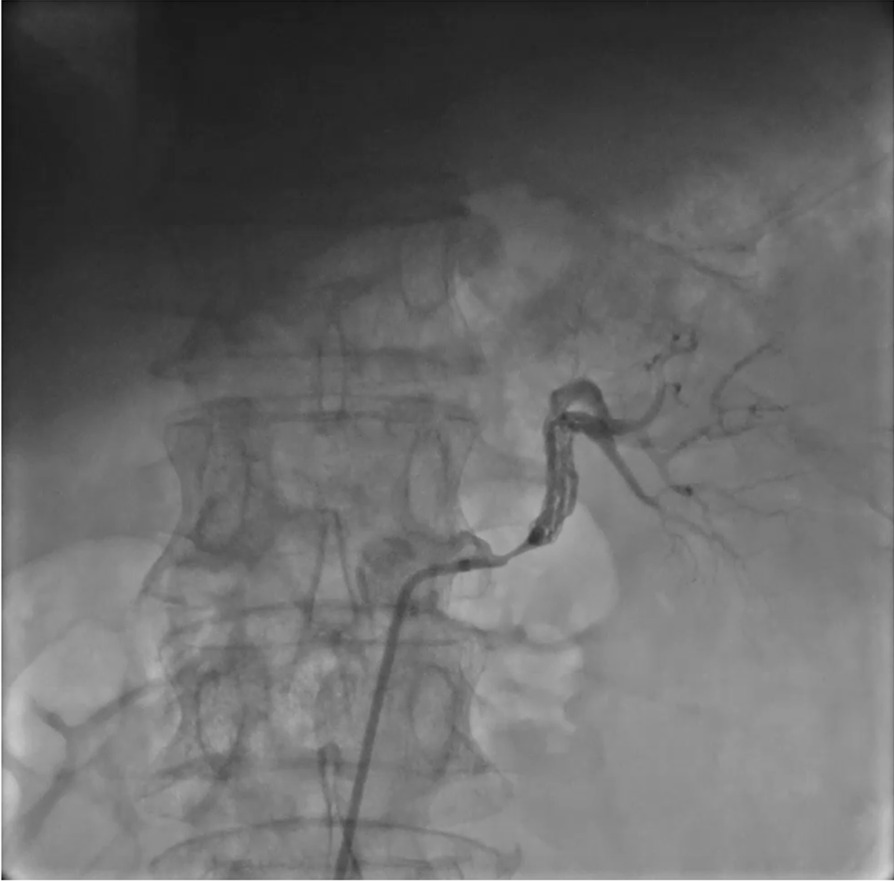
The CAG data on 12th October 2020 showed a 95% stenosis of the proximal segment of left renal artery and the middle part was blurred with multi-channel-like blood flow

**Fig. 2:**
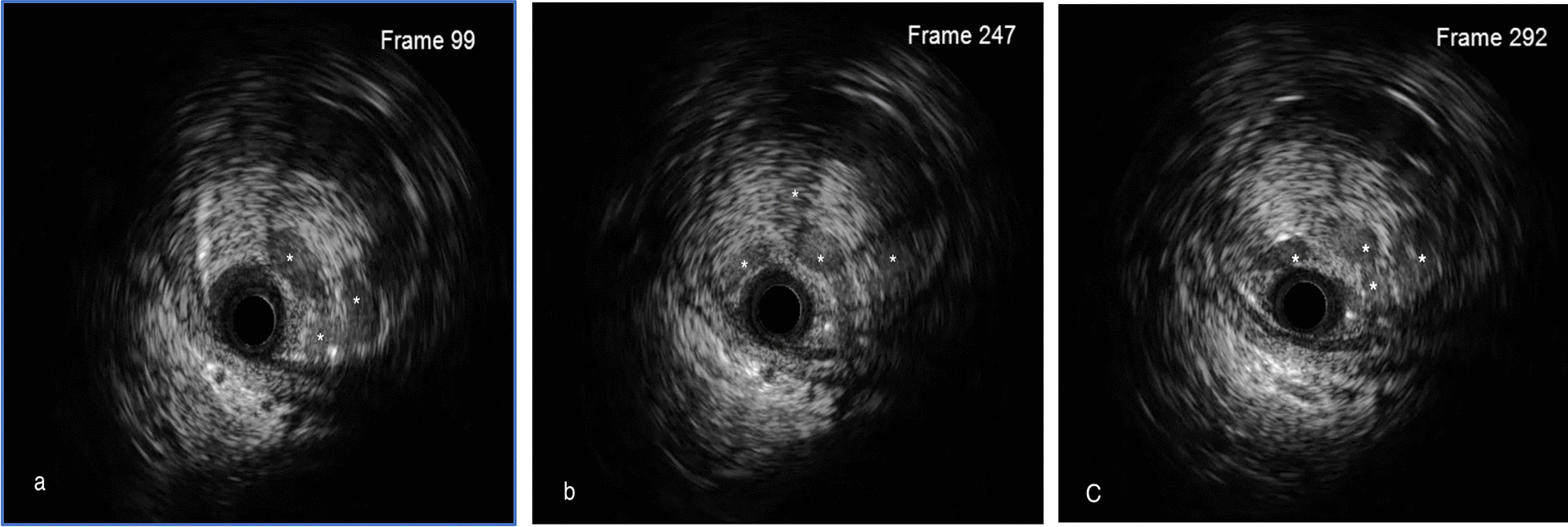
The IVUS finding (**a**–**c**). IVUS examination found multiple lumens in left renal artery (marked as *) and the cavity was filled with fibrous tissue

**Fig. 3:**
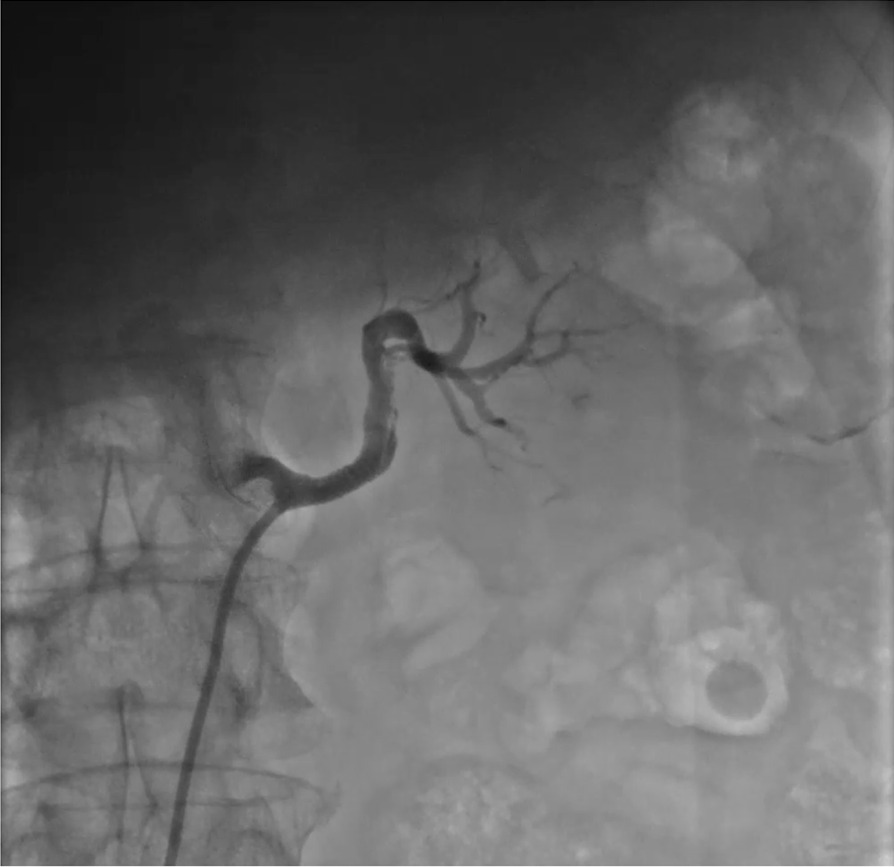
The CAG data after balloon dilatation and stent plantation. The final angiogram showed restored flow to distal renal artery

A 69-year-old Han man complained of chest distress and shortness of breath after stress for four months. He was diagnosed with hypertension for four months, and he felt that these symptoms were accompanied by high blood pressure. The maximum blood pressure measured was 200/110 mmHg. Benidipine hydrochloride (4 mg twice daily), metoprolol succinate (47.5 mg Qd), furosemide (20 mg Qd) and spironolactone (20 mg Qd) were applied to control blood pressure. In addition, he had a dull pain in his left waist for four months. Abdominal enhanced computerised tomography (CT) demonstrated a suspected left renal infarction. His past medical history included 10 years of diabetes mellitus and hyperlipidemia. During this hospitalization, he was diagnosed with resistant hypertension with chronic renal disease and renal dysfunction (creatinine 122 µmol/L, eGFR = 51.80 mL/min × 1.73 m^2^). Abdominal enhanced CT was reperformed and showed that his left renal artery was nearly occluded, his right renal artery had mild to moderate stenosis and his left kidney had atrophied. An invasive angiography with angiographic catheter JR 4.0 further demonstrated a 95% stenosis of the proximal segment of the left renal artery, and the middle segment was blurred with multi-channel-like blood flow (Fig. 1, Additional file 1). An RDC guiding catheter was used and run through across the lesion. The proximal lesion was pre-dilated by a 4.0 mm × 15 mm balloon at 10 atm. A commercially available IVUS system (iLAB, Boston Scientific Corporation, Marlborough, Massachusetts) was used to acquire IVUS images. A 40 MHz, 2.6 F imaging catheter (Atlantis SR Pro or Pro 2, Boston Scientific) was advanced distal to the lesion, and an automated pullback was performed at a speed of 0.5 mm/s. Multiple lumens and HLS were demonstrated by IVUS, and the cavity was surrounded by fibrous tissue (Fig. 2, Additional file 2). A lesion of the middle segment of the left renal artery was sequentially dilated by a 1.5 × 15 mm balloon, 2.5 × 10 mm balloon and 4.0 × 15 mm balloon at 8–12 atm, and an express SD 5.0 × 19 mm stent was implanted at 12 atm successfully, overlapping with the proximal segment of the left renal artery (Fig. 3, Additional file 3). The final angiogram showed restored flow to the distal renal artery. The patient recovered well and was discharged with atorvastatin (20 mg Qd), ezetimibe (10 mg Qd), aspirin (100 mg Qd), clopidogrel (75 mg Qd), Sacubitril Valsartan Sodium (25 mg Bid) and metoprolol succinate (47.5 mg Qd). Over three months of follow-up, the patient achieved complete remission of chest distress, shortness of breath and waist pain. Blood pressure was well controlled around 130/75 mmHg and renal function was recovered (creatinine 96 µmol/L, eGFR 69.21 mL/ (min × 1.73 m^2^).

## Discussion and conclusions

HLS is considered a rare vascular lesion with a diffuse lesion or a braid-like appearance that can be detected by angiography, and further intravascular examination, such as IVUS and coherence tomography (OCT), helped to confirm the multiple intraluminal channels or honeycomb structure, which can also be described as having a ‘swiss cheese-’, a ‘spider web-’, or a ‘Lotus root-like’ appearance [4]. IVUS and OCT are helpful and essential for diagnosing HLS [5]. HLS was mostly reported in the coronary artery, but there were also some case reports of HLS being located in the carotid artery and renal artery [3, 5, 6]. The aetiology of HLS is still not clear. Previous studies have reported that it can be caused by spontaneous recanalisation after thrombotic events, including in situ thrombosis and embolic thrombus. Thrombosis can be secondary to vasculitis caused by Kawasaki disease, antiphospholipid syndrome and vasospasm [1, 7–10]. With the application and development of intravascular examination, more and more HLS was found, and most of its causes were thought to be recanalisation of the in situ thrombus and embolic thrombus [5]. Kazuoki et al. reported serial coronary angiography of a post-emergent coronary artery bypass grafting patient and found that occlusive thrombus in the left circumflex artery (LCX) was recanalised spontaneously after two years of follow-up. A lotus-root appearance was then confirmed by OCT, which provided the direct evidence that the multiple communicating channel structure represented spontaneous recanalised lesions after the thrombotic occlusions [11]. A similar manifestation was also found in an effort angina patient and confirmed by high-resolution intravascular ultrasound (HR-IVUS) [12]. Despite recanalization, most HLS lesions significantly affect blood flow, with an FFR value < 0.8 [2]. Therefore, positive intervention therapy was suggested for HLS lesions. Previous studies reported different treatment strategies, including drug-eluting stent (DES) implantation, drug-coating balloon and bioresorbable vascular scaffold [5, 13, 14]. However, stent implantation, which compresses both the septa and cavities, may be associated with side branch compromise or occlusion [2].

To our knowledge, this is the first case of HLS on the basis of atherosclerosis in the renal artery. The renal artery multiple luminal surrounded by fibrous tissue was demonstrated by IVUS, and no intact vascular wall of branches was shown in intravascular imaging. The properties of the lesions could not be fully identified. Upon consideration of multiple atherosclerotic risk factors, including age, diabetes and hyper-lipidaemia, we surmised that the stenosis of the left proximal renal artery resulted from atherosclerosis. Based on serious stenosis of the proximal artery, the decreased velocity of distal blood flow led to thrombus formation, which was associated with renal infarction. The thrombus was recanalized and finally formed HLS in his renal artery as a result of mismanagement in the acute phase. In addition, three months of follow-up showed that the patient had no complaint after stent implantation, which also verified our previous speculations.

In conclusion, the mechanism and formation of HLS are still unclear. This malformation can be found not only in the coronary artery but also in the renal artery. Detailed patient histories, long-term follow-up, angiogram, and intravascular imaging data may offer a deeper understanding of HLS in future clinical practice.

## Supplementary Information

**Additional file 1**. Pre-operation renal artery arteriography.

**Additional file 2.** IVUS for honeycomb-like structure in left renal artery.

**Additional file 3**. Post-operation renal artery arteriography.

## Data Availability

All data generated or analyzed during this study are included in this manuscript and its additional files.

## Abbreviations

HLS: Honeycomb-like structure
LAD: Left anterior descending artery
CT: Computerized tomography
IVUS: Intravascular ultrasound
OCT: Coherence tomography
LCX: Left circumflex artery
HR-IVUS: High-resolution intravascular ultrasound
DES: Drug-eluting stent
